# Multimodal Fusion Prediction of Radiation Pneumonitis via Key Pre-Radiotherapy Imaging Feature Selection Based on Dual-Layer Attention Multiple-Instance Learning

**DOI:** 10.3390/jimaging12040158

**Published:** 2026-04-08

**Authors:** Hao Wang, Dinghui Wu, Shuguang Han, Jingli Tang, Wenlong Zhang

**Affiliations:** 1 School of Automation and Intelligent Sciences, Jiangnan University, Wuxi 214122, China; haowang@stu.jiangnan.edu.cn (H.W.); 6231905049@stu.jiangnan.edu.cn (J.T.); 2 Department of Respiratory and Critical Care Medicine, Wuxi School of Medicine, Jiangnan University Medical Center, Wuxi 214122, China; wenlongkekexili@163.com

**Keywords:** radiation pneumonitis, dual-layer attention, bag embedding, multiple-instance learning, multimodal

## Abstract

Radiation pneumonitis (RP), one of the most common and severe complications in locally advanced non-small cell lung cancer (LA-NSCLC) patients following thoracic radiotherapy, presents significant challenges in prediction due to the complexity of clinical risk factors, incomplete multimodal data, and unavailable slice-level annotations in pre-radiotherapy CT images. To address these challenges, we propose a multimodal fusion framework based on Dual-Layer Attention-Based Adaptive Bag Embedding Multiple-Instance Learning (DAAE-MIL) for accurate RP prediction. This study retrospectively collected data from 995 LA-NSCLC patients who received thoracic radiotherapy between November 2018 and April 2025. After screening, Subject datasets (*n* = 670) were allocated for training (*n* = 535), and the remaining samples (*n* = 135) were reserved for an independent test set. The proposed framework first extracts pre-radiotherapy CT image features using a fine-tuned C3D network, followed by the DAAE-MIL module to screen critical instances and generate bag-level representations, thereby enhancing the accuracy of deep feature extraction. Subsequently, clinical data, radiomics features, and CT-derived deep features are integrated to construct a multimodal prediction model. The proposed model demonstrates promising RP prediction performance across multiple evaluation metrics, outperforming both state-of-the-art and unimodal RP prediction approaches. On the test set, it achieves an accuracy (ACC) of 0.93 and an area under the curve (AUC) of 0.97. This study validates that the proposed method effectively addresses the limitations of single-modal prediction and the unknown key features in pre-radiotherapy CT images while providing significant clinical value for RP risk assessment.

## 1. Introduction

Lung cancer is one of the most common malignant tumors in the world, with its incidence and mortality rates ranking first among all cancers [1]. Radiation therapy, a cornerstone of cancer treatment, is used to treat or palliate 50% of cancer patients and is increasingly employed in the management of thoracic malignancies [2]. At various stages of primary lung cancer, approximately two-thirds of patients require radiotherapy [3]. Among the complications arising from thoracic radiotherapy, radiation-induced lung injury (RILI) is one of the most frequent and severe, affecting up to 20% of patients [4]. RILI manifests primarily as early radiation pneumonitis (RP) and late radiation pulmonary fibrosis (RPF). Currently, no specific treatment exists for RP or subsequent pulmonary fibrosis, making it crucial to find ways to prevent or mitigate RP during lung cancer radiotherapy.

Early diagnosis and prediction of RP are critical for lung cancer patients. They enable early assessment of high-risk patients and provide personalized treatment plans. But conventional diagnostic methods rely on clinical symptoms, imaging findings, and laboratory tests, all of which are subjective. These methods are limited by the variability in symptom presentation, the differences in radiologist interpretations, and the inconsistencies in laboratory results. In clinical practice, volume dose parameters derived from dose–volume histograms (DVHs) are commonly used to predict RP. However, this approach only considers the dose distribution and lacks detailed spatial information about lung tissue, potentially overlooking critical areas that are susceptible to injury. Furthermore, discrepancies in DVH calculations between different systems can undermine the reliability of RP predictions. With advances in digital imaging and storage technologies, deep learning has become a promising tool for RP prediction. Prediction models can be categorized as unimodal or multimodal, with the latter integrating multiple data sources (clinical data, imaging features, and treatment parameters) into a unified framework, offering a more comprehensive analysis than traditional unimodal models. In unimodal studies, Zhang et al. [5] developed a radiomics model using computed tomography (CT) images to predict RP. They extracted radiomics features from CT images and employed a support vector machine (SVM) algorithm, achieving an area under the receiver operating characteristic curve (AUC-ROC) of 0.89 and demonstrating the potential of radiomics for RP prediction. Raptis et al. [6] proposed a deep learning-based radiomics model that extracted key features from radiographic images to improve RP prediction performance. However, relying on a single modality limits the generalizability of the model. In multimodal studies, Lee et al. [7] integrated data from chest CT scans, clinical factors, and other variables using a MergeNet neural network, but the model’s performance was suboptimal and not yet suitable for clinical use. Zhang et al. [8] proposed a deep learning model combining CT and radiation dose (RD) images to predict RP in lung cancer patients undergoing radical (chemo)radiotherapy. Kawahara et al. [9] developed an RP prediction model using a convolutional neural network (CNN) with image cropping and deep learning based on 3D CNNs. However, their model did not account for clinical factors such as age, chemotherapy, and smoking history, and its predictive robustness was limited. Ai et al. [10] proposed a method that integrates radiomics, deep learning features, dosiomic features, and SUVp90 for radiation pneumonitis prediction, in which Extreme Gradient Boosting (XGBoost) and ResNet-18 achieved the best performance. Kong et al. [11] developed a prediction model for symptomatic radiation pneumonitis by combining imaging features extracted from radiotherapy planning CT using a 3D deep learning network with dose–volume metrics. However, these studies did not consider the impact of non-key instances (slices) on the performance of the prediction model.

In actual model training, a large number of medical samples are needed and require professional annotation, whose labeling accuracy will directly affect the diagnostic results of the model. In addition, instance-level labeling not only consumes a lot of human and material resources but also causes certain difficulties in data collection [12]. Therefore, related scholars have used the concept of multi-instance learning (MIL) to solve the problem of accurate sample labeling [13,14]. The concept of MIL was first proposed by Dietterich et al. [15] to solve the drug activity prediction. In their methodology, it is elaborated that the MIL framework is a technique to overcome the limitations of traditional supervised learning when single-instance labeling is not available to deal with weakly supervised problems. In the MIL idea, labels are not assigned to individual instances (samples). Instead, instances (samples) with the same label are grouped into packages, where each package can contain a different number of instances. When assigning labels to each package, the package is labeled positively if at least one instance exists in the package and negatively if the package is full of negative instances.

Due to its ability to simplify dataset construction and reduce annotation workload, MIL has been widely applied in medical image analysis, including whole-slide imaging (WSI), X-ray, and CT. Recently, several MIL algorithms, such as Diverse Density (DD), EM-DD [16], MI-SVM, and mi-SVM [17], have been proposed. For example, Zhu et al. [18] modeled lung cancer image classification as a multi-class MIL problem, where cellular features from lung cancer images were extracted as bags, transformed using the Hausdorff distance, and classified using an AdaBoost algorithm. Xu et al. [19] proposed a multi-clustering MIL (MCIL) method for histopathology image segmentation and classification, embedding clustering concepts into MIL to reduce ambiguity. Li et al. [20] introduced an MIL method for determining tumor infiltration depth in gastric cancer using dual-energy CT, extracting both bag-level and instance-level features for classification.

**Figure 1:**
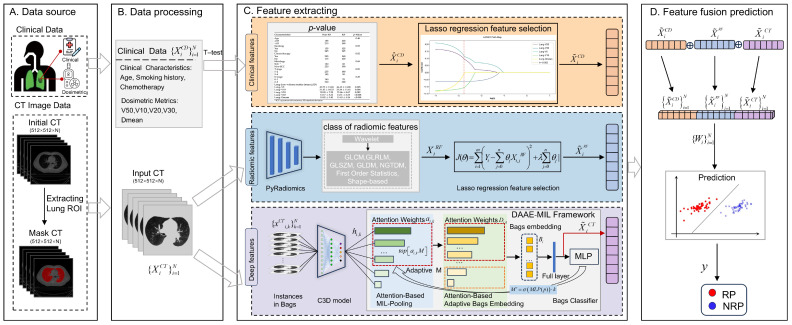
The overall workflow of the radiation pneumonitis predictive model. The pipeline consists of four main components: (**A**) data sources, (**B**) data processing, (**C**) feature extraction, and (**D**) feature fusion and prediction.

This study focuses on predicting RP, a common adverse event in locally advanced non-small cell lung cancer (LA-NSCLC) patients undergoing radiotherapy, by utilizing pre-treatment CT images and clinical data. However, RP prediction faces challenges including the complexity of clinical pathogenic factors and unavailable instance-level annotations in pre-radiotherapy CT images. To address these issues, we propose a multimodal model with deep MIL for RP prediction. The model aims to reduce interference from non-critical slice-level features, focusing on key instance features, while also integrating multi-modal data to enhance prediction accuracy. Specifically, the model first extracts features from 3D CT imaging data using a fine-tuned C3D network. Next, it employs the Dual-Layer Attention-Based Adaptive Bag Embedding MIL (DAAE-MIL) algorithm to select highly correlated instance features and generate instance bags containing key instance features to obtain deep CT features. Finally, the CT deep features are fused with clinical and radiomics features to achieve precise prediction of RP. The framework of the model is shown in Figure 1. The main contributions of this paper are as follows:

(1) Addressing the Unavailable Slice-Level Annotations in Pre-radiotherapy Datasets: To tackle the challenge of unavailable slice-level annotations in pre-radiotherapy CT images, we propose a DAAE-MIL model. This model leverages a dual-layer attention mechanism to screen critical instance features and generate bag-level representations, effectively reducing interference from non-critical slice-level features while focusing on the extraction of discriminative instance features.

(2) Enhancing Radiomics-Based Prediction of Radiation Pneumonitis. To improve the accuracy of RP prediction, we introduce a multimodal prediction framework that integrates deep features from CT images, clinical data, and radiomics features. This framework significantly addresses the limitations of single-modal prediction models, achieving superior performance by capturing complementary information across modalities.

(3) Validation and Ablation Studies: The proposed framework is extensively validated on a proprietary dataset, demonstrating its superior predictive performance in RP classification tasks. Ablation studies further confirm the effectiveness of each proposed module, highlighting their individual contributions to the overall model performance.

## 2. Materials and Methods

### 2.1. Data

In this study, a retrospective collection of data involving 995 LA-NSCLC patients who received thoracic radiotherapy at the JUMC Hospital was obtained from November 2018 to April 2025. This dataset includes CT images from 270 patients with radiation pneumonitis and 725 patients without radiation pneumonitis (NRP). The requirement for written informed consent was waived due to the characteristics of this retrospective study. To avoid any potential breach of patient confidentiality, the data were deidentified and had no link to research. The inclusion criteria were as follows:

(a) Pathological confirmation of unresectable LA-NSCLC, generally through multidisciplinary discussions, receiving chest conventionally fractionated intensity-modulated radiotherapy (IMRT) with or without platinum-based chemotherapy.

(b) An Eastern Cooperative Oncology Group (ECOG) performance score of ≤2 before radiotherapy, without interstitial lung disease or severe heart, liver, or kidney dysfunction.

(c) Follow-up of more than 6 months after the completion of radiotherapy.

(d) Patients with complete pre-radiotherapy CT images, clinical variables, and key dosimetric records.

In this study, two experienced radiation oncologists conducted monthly follow-ups for symptomatic RP within 6 months after radiotherapy and evaluated the condition based on CT images and clinical information before and after follow-up. RP grading was independently performed by the two radiation oncologists according to the acute RP grading standard of the Radiation Therapy Oncology Group (RTOG). In cases of disagreement, the final grade was determined through joint review and discussion with reference to the complete clinical records and imaging findings. Symptomatic RP was defined as grade ≥2.

The CT scans were obtained using a GE LightSpeed Ultra CT scanner. The CT scanning parameters were obtained with a tube voltage of 120 kVp, automatic tube current modulation (mAs), a matrix size of 512×512, a thickness of 5 mm per layer, and a high-spatial-resolution algorithm. All patients were treated with IMRT using 6 MV X-rays from linear accelerators, and the median total prescribed dose was 60 Gy (range: 54–66 Gy) with a median fractional dose of 2 Gy (range: 1.8–3 Gy). In this study, the regions of interest (ROIs) were defined on the pre-radiotherapy CT as bilateral lungs excluding the planning target volume (PTV) (Lung-PTV), with exclusion of hilar and atelectasis, as well as thickening of the pleura. Delineation was performed by a junior radiologist and reviewed by an experienced radiologist.

### 2.2. Data Preprocessing

In this paper, the research goal is to extract instance-level features from samples that only have package-level annotations, learn the relationship between packages and key samples through an MIL-based multimodal model, and integrate data across different domains (clinical data and radiomics features) to achieve high classification performance. In the case of only unique CT data and clinical data for each patient, set $X=\left\{X_{1},X_{2},\ldots ,X_{N}\right\}$ to denote the samples of N patients and their corresponding tag sets ($Y=\left\{Y_{1},Y_{2},\ldots ,Y_{N}\right\}$, $Y_{N}\in \left\{0,1\right\}$). In this study, the RP ≥2 level was identified as a case of RP, and the samples were labeled as 1 accordingly and 0 otherwise.

#### 2.2.1. Clinical Data

The collected sample clinical dataset ${\left\{X_{i}^{CD}\right\}}_{i=1}^{N}$ consisted of attributes such as gender, age, radiotherapy dose ($V_{5}\;\mathrm{Gy},\;V_{10}\;\mathrm{Gy},\;V_{20}\;\mathrm{Gy},\;V_{30}\;\mathrm{Gy}$), and smoking history of the patients, which were analyzed using one-way analysis of the correlation of clinical and dosimetric parameters with the occurrence of radiation pneumonitis and initially screened for potential factors predictive of pneumonitis (p≤0.05 was considered to be statistically significant). For sample $X_{i}^{CD}\;=\;\left(x_{i,1}^{CD},x_{i,2}^{CD},\ldots ,x_{i,n}^{CD}\right)$, $x_{i,n}^{CD}$ represents the n-th clinical characteristic of the i-th sample. To investigate the differences in characteristics between RP and non-RP cases ($Y_{N}\in \left\{0,1\right\}$), statistical analyses were performed accordingly: the independent-samples *t*-test was utilized for continuous variables (such as dosimetric metrics), whereas the Chi-Square test was applied to categorical variables (such as smoking history, histology, and chemotherapy status). A correlation threshold (α=0.05 was considered statistically significant) was set for the screening of potential factors, resulting in the following set of significantly correlated features:

(1) $$T_{sig}=\left\{n|p\text{-}\mathrm{value}\left(x_{i,n}^{CD}\right)<\alpha \right\}$$

In order to reduce the feature dimensionality of the clinical data, the clinical and dosage data were normalized using a Z-score prior to Lasso regression, which transformed the feature values into a distribution with a mean of zero and a standard deviation of one. In order to identify significantly correlated features, Lasso regression of the statistically screened features was required, aiming to minimize the following objective function:

(2) $$\begin{array}{c} \hat{\beta }=\arg\min_{\beta }{\left\{\frac{1}{2M}\sum_{m=1}^{M}(Y_{N}-\beta_{0}-\sum_{n\in T_{sig}}x_{i,n}^{CD}\beta_{n}\right)}^{2}+\lambda \sum_{n\in T_{sig}}\left|\beta_{n}\right|\} \end{array}$$

where $\beta_{0}$ is a constant term, $\beta_{n}$ is the regression coefficient of the nth feature, and λ is the regularization parameter. The index set $T_{LASSO}$ of the non-zero features is chosen:

(3) $$T_{LASSO}=\left\{n\in T_{sig}|{\hat{\beta }}_{n}\neq 0\right\}$$

The filtered features can be expressed as

(4) $${\tilde{X}}_{i}^{CD}={\left(x_{i,n}^{CD}\right)}_{n\in T_{LASSO}}$$

The determination of the optimal λ is a critical step in LASSO-based feature selection. An excessively large λ may shrink most coefficients to zero, resulting in an overly simplified model, whereas an overly small λ may retain too many variables and lead to overfitting. In this study, cross-validation is commonly employed to address this issue by randomly partitioning the dataset into K subsets and computing the mean squared error (MSE) corresponding to each λ. The λ that yields the lowest average validation error is then selected as the optimal regularization parameter.

#### 2.2.2. Radiomics Features

Radiomics, as an emerging medical image analysis technique, uses a variety of statistical analyses and data mining methods to extract high-throughput features from regions of interest in images. In this paper, radiomics features (e.g., texture, shape and size) were extracted using the open-source Python library PyRadiomics (version 3.0.1, https://pyradiomics.readthedocs.io/en/latest/ (accessed on 27 February 2025)).

A total of 758 radiomics features were extracted form the ROI of each CT (Lung-PTV). These features were categorized into four groups: (1) 14 shape features; (2) 18 first-order features; (3) texture features, namely 24 Gray-Level Co-occurrence Matrices (GLCMs), 16 Gray-Level Run Length Matrices (GLRLMs), 16 Gray-Level Size Zone Matrices (GLSZMs), 5 Neighbouring Gray Tone Difference Matrix (NGTDM) features, and 14 Gray-Level Dependence Matrices (GLDMs), as well as shape features and first-order features; and (4) wavelet filtering, which yields 8 decompositions per level (all possible combinations of applying either a high- or low-pass filter in each of the three dimensions). We then filter the first-order features and texture features extracted from the original image to obtain the wavelet-filtered features. Radiomics features are expressed as

(5) $${X_{i}}^{RF}=PyRadiomics\left({X_{i}}^{CT}\right)$$

where, PyRadiomics(·) represents the radiomics feature extraction function, and the extracted features can be expressed as

(6) $${X_{i}}^{RF}=\left(x_{i,1}^{RF},x_{i,2}^{RF},\ldots ,x_{i,n}^{RF}\right)$$

After extracting radiomics features, it is also necessary to preprocess the data using Z-score normalization and to perform feature selection with the Lasso model. Similarly, the index set $J_{LASSO}$ of the non-zero features is selected as follows:

(7) $$J_{\mathrm{LASSO}}=\left\{j\in \left\{1,2,\ldots ,n\right\}|{\hat{\beta }}_{j}\neq 0\right\}$$

where ${\hat{\beta }}_{j}$ is the regression coefficient of the jth feature. The radiomics features after screening were

(8) $${\tilde{X}}_{i}^{RF}={\left(x_{i,n}^{RF}\right)}_{n\in J_{\mathrm{LASSO}}}$$

#### 2.2.3. CT Deep Features

In this paper, the patient CT image dataset ${\left\{X_{i}^{CT}\right\}}_{i=1}^{N}$ uses Lung-PTV as the ROI, and it is delineated by experienced doctors. Sample $X_{i}^{CT}$ is a 3D CT image with dimensions of H×W×S, where *H* and *W* represent the height and width (512 and 512), as well as the depth (number of slices) *S* (S=k). Before inputting the data into the deep feature extraction model, it needs to be preprocessed. In this paper, the CT image is processed by Min-Max normalization so that the gray value of the voxel is linearly adjusted to the range of [−1, 1]. The normalized sample $X_{i,norm}^{CT}$ is defined as follows:

(9) $$X_{i,norm}^{CT}=\frac{X_{i}^{CT}-\min\left(X_{i}^{CT}\right)}{\;\max\left(X_{i}^{CT}\right)-\min\left(X_{i}^{CT}\right)}$$

### 2.3. Phantoms

During the collection of clinical radiomics data, slice-level label data is often challenging to obtain, which can interfere with feature extraction by deep network models and impact their predictive accuracy. To address this issue, this study proposes a DAAE-MIL model using patient-level label data, thereby reducing the model’s reliance on slice-level labels. First, a fine-tuned C3D model is introduced to extract deep CT features. Next, the extracted features are fed into the DAAE-MIL model, where a bi-level attention mechanism selects key instances to embed into the instance bags, ultimately obtaining the deep features of the key instances.

#### 2.3.1. Multiple-Instance Learning Theory

MIL is a form of weakly supervised learning where multiple instances are aggregated into a single instance bag, with the bag assigned a corresponding label, while the labels of individual instances within the bag remain unknown. Given the high diagnostic significance of 2D axial slices in 3D medical imaging data, MIL can be effectively applied in weakly supervised scenarios to predict RP from 3D medical imaging data.

In MIL algorithms [21,22], each instance $x_{i,k}^{CT}$ is assumed to have an instance label $y_{i}=\left\{0,1\right\}$. For a given bag of instances, not all instances are purely positive or purely negative, nor do they necessarily contribute equally to the learning process. Thus, a bag is assigned a negative label if all instances within it are negative; otherwise, it is assigned a positive label. This relationship is governed by the following MIL constraint:

(10) $$Y=\left\{\begin{array}{l} 0,iff\sum_{N}Y_{N}=0\\ 1,otherwise \end{array}\right.$$

Based on the MIL constraints, instance bags are constructed from the patient CT data sample set ${\left\{X_{i}^{CT}\right\}}_{i=1}^{N}$, where each patient’s sample $X_{i}^{CT}$ forms an instance bag with a corresponding label set $Y=\left\{Y_{1},Y_{2},\ldots ,Y_{N}\right\}$. Here, $Y_{N}\in \left\{0,1\right\}$ represents RP or non-RP labels. Each 2D axial slice $\left\{x_{i,1}^{CT},x_{i,2}^{CT},\ldots ,x_{i,k}^{CT}\right\}$ is treated as an instance embedded in the bag, and the embedded instances from the 3D CT data are aggregated to form the bag.

**Figure 2:**
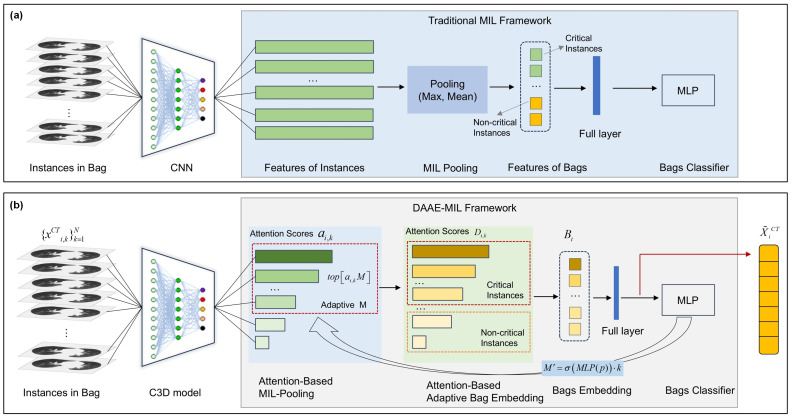
Deep feature extraction network based on MIL. (**a**) The conventional MIL network first extracts instance features using a CNN, then inputs them into an MIL pooling layer to perform bag-level feature aggregation, and finally classifies the aggregated representation. (**b**) The proposed DAAE-MIL network extracts both instance features and sequential features using C3D, then applies a dual-layer attention mechanism for feature selection, forming a highly relevant instance aggregation.

Current deep feature extraction models based on MIL predominantly focus on processing individual instances in isolation, thereby neglecting the critical spatial and sequential correlations between adjacent CT scan slices. The convolutional neural network (CNN) is commonly employed as the feature extraction model, as illustrated in Figure 2a. A representative example is the ResNet series (ResNet10 101), which is composed of several basic blocks, each containing a skip connection. Each block consists of two convolutional layers with a kernel size of 3×3, followed by batch normalization and a rectified linear unit (ReLU) activation function. However, ResNet is inherently limited in its ability to capture sequential information across slices. To address this fundamental limitation, we propose a fine-tuned C3D network as the spatiotemporal-aware feature extractor, specifically designed to preserve inter-instance spatial and volumetric contextual information. The C3D network’s input channels are adjusted to 1, and the original model’s final pooling layer and fully connected layers are removed. The fine-tuned C3D model structure serves as a feature extractor without a classifier.

The 3D CT sample $X_{i}^{CT}$ adaptively extracts slices ${\left\{x_{i,k}^{CT}\right\}}_{k=1}^{N}$ as input, and after passing through the final convolutional layer, it outputs a series of feature maps $H_{i}\in R^{H\times W\times S}$, where the number of features is determined by the number of channels in the last layer. As a feature extractor, the model is capable of adapting to the CT image inputs from the dataset used in this study and extracting the deep features of instances. The network structure is illustrated in Figure 2, and the feature extraction process can be expressed as

(11) $$H_{i}=f\left(X_{i}^{CT}\right)$$

where f(·) represents the instance-level deep feature extractor. The output of the model, denoted as $H_{i}=\left\{h_{i,1},h_{i,2},\ldots ,h_{i,k}\right\}$, is a set of deep feature vectors that form the subsequent input. For each instance, the mapped feature vector is represented as $h_{i,k}=f\left({x^{CT}}_{i,k}\right)\in R^{HW\times 1}$, k=HW.

#### 2.3.2. Dual-Layer Attention-Based Adaptive Bag Embedding Framework (DAAE)

**Figure 3:**
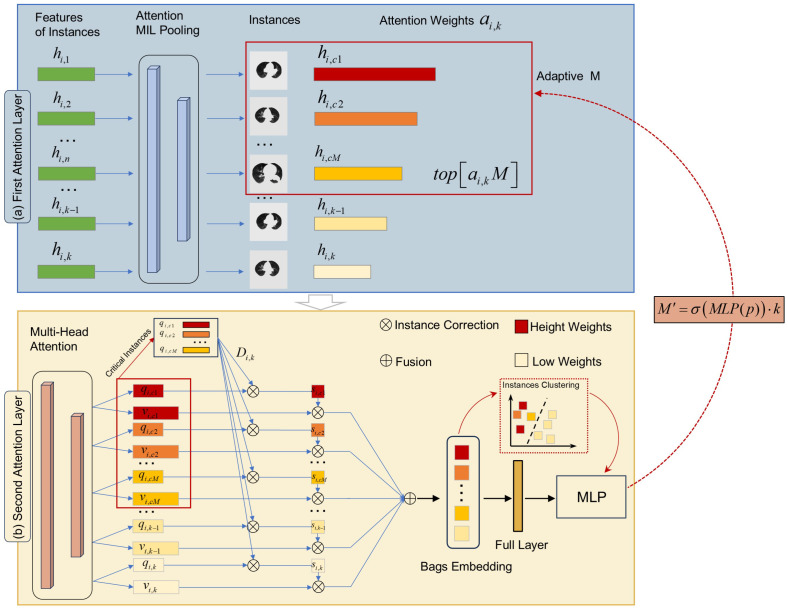
Dual-Layer Attention-Based Adaptive Bag Embedding (DAAE) framework. The framework comprises two hierarchical attention layers: (**a**) an attention-based MIL-pooling layer that scores and weights instance-level features and (**b**) a multi-head attention layer that performs feature selection and generates bag-level embeddings.

According to the definition of MIL in Section 2.3.1, negative bags exclusively contain negative instances, whereas positive bags encompass both positive and negative instances. However, most existing single-layer attention-based MIL methods perform a simple weighted summation over all instances, where noisy or irrelevant negative instance slices can still degrade the model’s predictive performance and robustness. To address this limitation, we propose a Dual-Layer Attention-Based Adaptive Bag Embedding (DAAE) Model (Figure 3), which filters critical instances from positive bags and dynamically embeds them into bags, thereby enhancing the precision of downstream predictive models. Figure 2 presents a comparison between conventional MIL methods and the proposed approach.

Attention-based MIL pooling: Most existing studies have predominantly employed two conventional feature aggregation approaches: max pooling and mean pooling for MIL-based frameworks. However, their simplistic mechanisms often result in suboptimal performance due to insufficient discriminative power. To address this limitation, we propose an attention-based MIL-pooling method that leverages an attention-driven mechanism to detect and prioritize critical instances within bags.

Based on the aforementioned feature extraction method, the feature vector of each instance $h_{i,k}=f\left({x^{CT}}_{i,k}\right)\in R^{HW\times 1}$ is utilized as the input at this stage. These vectors are subsequently processed through the first-layer attention-based MIL-pooling method to generate the bag representations $Z_{i}$, along with their corresponding attention weights $a_{i,k}$.

(12) $$a_{i,k}=\frac{\exp\left\{w^{⊤}\tanh\left(Vh_{i,k}^{⊤}\right)\right\}}{\sum_{j=1}^{K}\exp\left\{w^{⊤}\tanh\left(Vh_{i,j}^{⊤}\right)\right\}}$$

where $w\in R^{L\times 1}$ and $V\in R^{L\times M}$ are learnable parameter matrices, and the hyperbolic tangent function $\tanh\left(\cdot \right)$ is used element-wise for non-linear transformations, which helps ensure proper gradient flow.

Attention-based adaptive bag embedding: The second-layer attention mechanism is designed to perform refined critical instance screening for final bag generation. Specifically, we first select the top-ranked instances based on the attention scores from the preceding pooling layer:

(13) $$top\left[a_{i,k}M\right]=\left\{h_{i,c1},h_{i,c2},\ldots ,h_{i,cM}\right\}$$

where *M* denotes the number of calibrated critical instances. Crucially, M is dynamically adjusted according to the model’s predictive confidence to achieve optimal alignment with the target task. This adaptive process is formulated as

(14) $$p=Soft\max(top-\left[a_{i,k}M\right])$$

(15) $$M^{\prime }=\sigma \left(MLP(p)\right)\cdot k$$

where *p* denotes the predicted probability distribution, *k* denotes the total number of candidate instances in the current bag, $\sigma \left(\cdot \right)$ denotes the sigmoid activation function constraining outputs to [0, 1], and $M^{\prime }$ is assigned to *M* to complete the parameter-adaptive update.

Secondly, the attention score for each instance is calibrated by calculating the relative average distance between the critical instance features. Based on the definition of multi-head attention, each instance is transformed into a query vector and an information vector.

(16) $$q_{i,k}=W_{q}h_{i,k}\&v_{i,k}=W_{v}h_{i,k},k=1,\ldots ,N$$

where $W_{q}$ and $W_{v}$ are the learnable weight matrices. The average relative distance between instance $h_{i,k}$ and key instance feature $h_{i,cM}$ is defined as $D_{i,k}$.

(17) $$D_{i,k}=\frac{1}{N}\sum_{m=1}^{M}\left(\frac{\exp\left(\langle q_{i,k},q_{i,cm}\rangle \right)}{\sum_{n=1}^{N}\exp\left(\langle q_{i,n},q_{i,cm}\rangle \right)}\right)$$

where $\langle \cdot ,\cdot \rangle$ is denoted as the inner product of the two vectors and $D_{i,k}$ is the second-layer attention weights, which are used as a threshold for generating bags.

Compared to conventional single-layer attention models that evaluate each instance in isolation, the inclusion of this second layer offers a significant structural advantage. By calculating the relative distance between candidate instances $q_{i,k}$ and the Top-M verified critical instances $q_{i,cm}$, the model ensures that high attention weights are only assigned to slices intrinsically correlated with the core diagnostic region. This dual-layer attention-based screening framework effectively suppresses the interference from noisy instances introduced by single-layer attention, thereby forming a more robust bag-level representation.

The bag embedding $B_{i}$ can be expressed as

(18) $$B_{i}=\sum_{k=1}^{N}D_{i,k}\cdot v_{i,k}$$

Finally, the depth features are obtained by extracting the last layer of the features as follows:

(19) $${{\tilde{X}}_{i}}^{CT}=Flatten\left(B_{i}\right)$$

#### 2.3.3. Multimodal Fusion and Prediction

Based on the research outlined above, features are extracted from three modalities: CT images, radiomics, and clinical data. After this dimensionality reduction step, the corresponding deep features (${\tilde{X}}_{i}^{CT}$) from CT images, radiomics features (${\tilde{X}}_{i}^{RF}$), and clinical data (${\tilde{X}}_{i}^{CD}$) are then fused using a cascading approach. This fusion involves concatenating the three different sets of features, which can be expressed as follows:

(20) $$W_{i}={\tilde{X}}_{i}^{CD}⊕{\tilde{X}}_{i}^{RF}⊕{\tilde{X}}_{i}^{CT}$$

where ⊕ denotes the concatenation of the feature vectors and $W_{i}$ represents the fused feature vector for the ith instance. Through this concatenation, a feature set with all three modalities (${\left\{W_{i}\right\}}_{i=1}^{N}$) is formed, and the fused features are used as input for a machine learning prediction model. In this study, a multilayer perceptron (MLP) is adopted as the prediction network model for RP. Its network structure consists of three layers and multiple neurons (also called perceptrons). The input layer is responsible for receiving signals; the hidden layer is responsible for computational tasks, and the output layer is responsible for making decisions based on input data. Each perceptron receives multiple input features, with each feature associated with a corresponding weight. The model is trained through forward propagation of signals and backward propagation of errors, which iteratively adjust and optimize the weights and biases. The output of a neuron, *a*, can be expressed as

(21) $$a=f\left(\sum_{i}w_{i}W_{i}+b\right)$$

where *f* is the Sigmoid activation function f(z)=1/(1+exp(−z)), $w_{i}$ represents the weights of the input features, $W_{i}$ represents the input values, and *b* is the bias term.

The final prediction result can be expressed as

(22) $$y=Model(W_{i})$$

### 2.4. Statistical Analysis

The experiments were conducted on a Windows 11 system equipped with an Intel Core i7-13700K CPU, 16 GB of RAM, and an NVIDIA RTX 3090 GPU (24 GB VRAM). All the aforementioned analyses were performed using Python. The models were implemented using Python 3.11.5 and PyTorch 2.1.2, with CUDA version 12.4. The training of model was optimized using the Adam optimizer with an initial learning rate of $1\times 10^{-4}$ and a weight decay of $1\times 10^{-5}$. The batch size was explicitly set to 4. The overall network was trained for up to 100 epochs, incorporating an early stopping strategy with a patience of 15 epochs based on the validation loss to prevent overfitting. The validity of the prediction models was evaluated using five-fold cross-validation (5-Fold CV) on the dataset, and statistical significance was established at *p* < 0.05. The evaluation metrics used in this study include accuracy, AUC, the F1-score, recall and precision. To strengthen the credibility of the reported performance, the 95% confidence intervals (CIs) for the AUC were calculated and reported, providing a rigorous statistical estimation of the model’s reliability across different test cohorts.

## 3. Results

A balanced distribution of positive and negative samples was maintained, thereby minimizing the impact on model performance. After screening, the present study ultimately included 670 patients from the collected dataset (995 patients) for experimental analysis. To rigorously evaluate the model and prevent any patient-level information leakage, the data-splitting strategy was strictly executed at the patient level. This ensured that all CT slices, clinical data, and radiomics features associated with a single patient were assigned entirely to either the training or testing cohort. Of these, 80% of the data were used for training (*n* = 535), and the remaining 20% were for testing (*n* = 135). The ratio of positive to negative samples is consistent in both the training and test sets, at 320:215 and 80:55 respectively. The training dataset was evaluated using five-fold cross-validation, while the test dataset was used as an independent test set.

### 3.1. Correlation Analysis and Feature Selection

**Figure 4:**
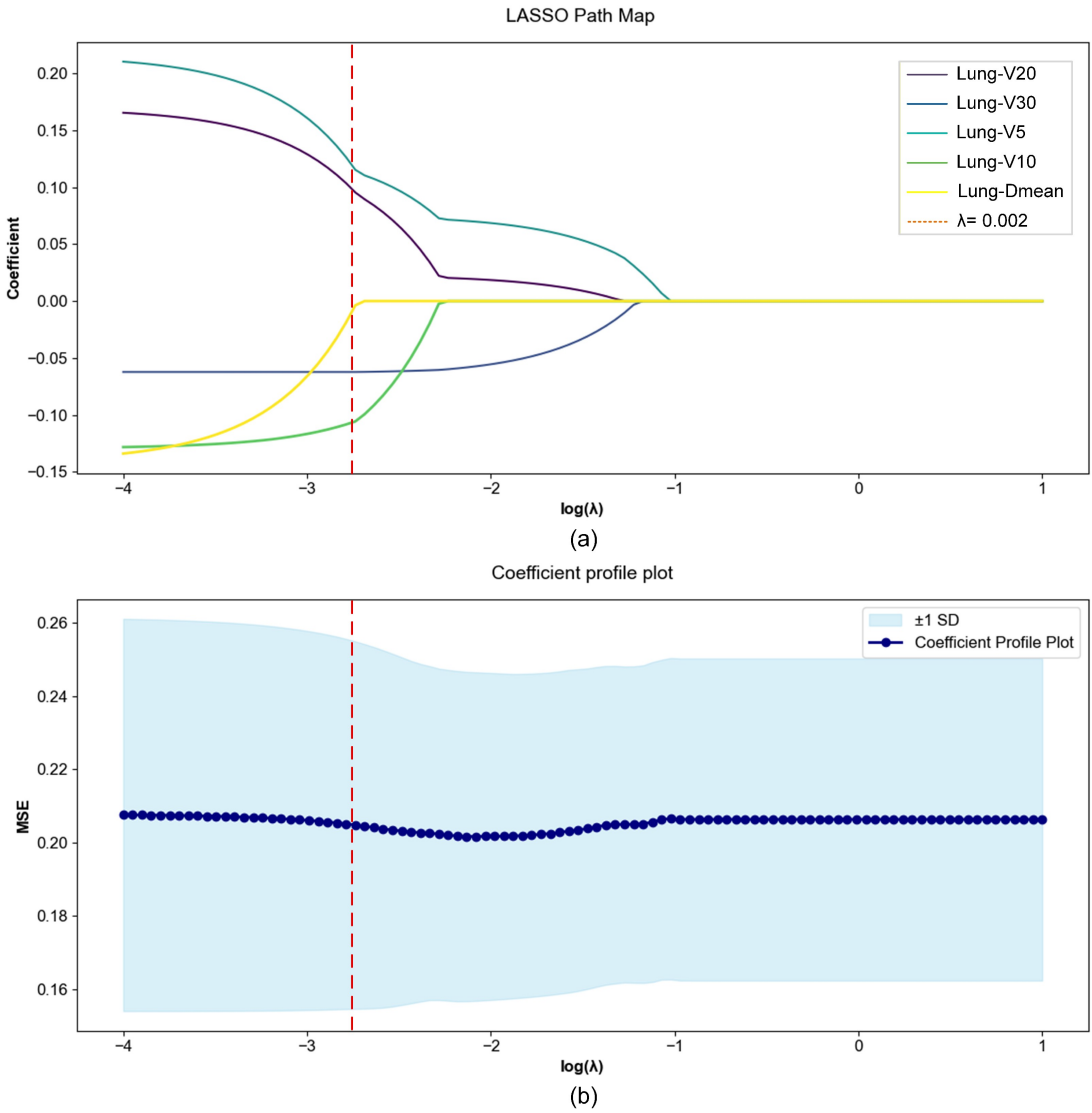
Procedures of clinical feature dimensionality reduction and selection based on LASSO. (**a**) LASSO path plot illustrating feature selection through regularization parameter (λ = 0.002) tuning. (**b**) Coefficient profile plot demonstrating the relationship between feature coefficients and the corresponding (λ = 0.002) values, reflecting dynamic changes in feature importance.

Clinical data: Table 1 presents the *p*-values used to evaluate the association between clinical and dosimetric parameters with the occurrence of RP. Continuous variables were analyzed using the independent *t*-test, while categorical variables were assessed using the Chi-Square test. The null hypothesis of the *t*-test assumes no significant difference in clinical and dosimetric characteristics between patients who developed RP and those who did not. The results of the univariate analysis indicate that clinical characteristics did not show statistically significant differences between the two groups (*p* > 0.05). However, significant differences were observed in dosimetric features, including V5 Gy, V10 Gy, V20 Gy, V30 Gy and Lung-Dmean (*p* < 0.05), suggesting a strong association between dosimetric parameters and RP occurrence. In light of these findings, LASSO regression analysis was further employed to identify key features from the dosimetric parameters. Based on the LASSO analysis in Figure 4, feature selection was performed by tuning the regularization parameter λ. In Figure 4a, the coefficient paths of dosimetric features are plotted against log(λ), showing that the coefficients are progressively shrunk toward zero as the regularization strength increases. Figure 4b presents the cross-validated mean squared error (MSE) curve with the corresponding variability band and the selected value (λ = 0.002), which provides a favorable trade-off between predictive error and model sparsity. At this λ value, five dosimetric variables (V5 Gy, V10 Gy, V20 Gy, V30 Gy, and Lung-Dmean) retained non-zero coefficients and were therefore selected for subsequent modeling.

**Figure 5:**
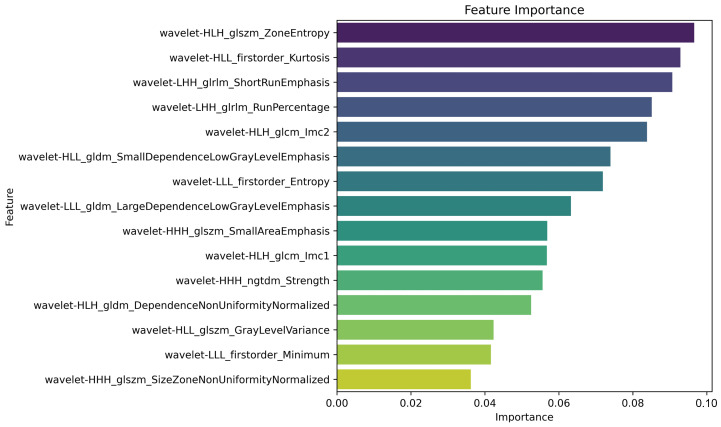
Radiomics feature dimensionality reduction and selection. Feature screening was performed based on the importance of features in response to LASSO regularization intensity.

Radiomics Features: Wavelet transformation was applied to the original images for denoising and filtering, resulting in the extraction of a total of 758 radiomics features for each ROI (Lung-PTV). According to the regularization intensity of LASSO, features of higher importance were selected. Figure 5 displays the feature importance plot, where LASSO selected 15 high-importance features.

**Figure 6:**
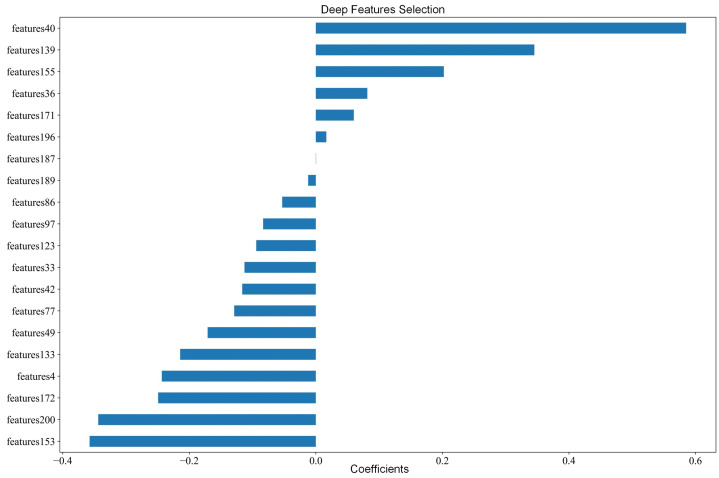
CT deep feature selection. The deep features with non-zero coefficients were selected.

Deep Features: Instance-level deep features were obtained from the last layer of the model. Due to the high dimensionality of these features, further feature selection was performed to reduce redundancy and enhance the model’s generalization capability. The LASSO regression algorithm was employed to screen 2048 deep features, ultimately selecting 21 features with non-zero coefficients. Specifically, Figure 6 visualizes the retained features with non-zero coefficients (*n* = 21). The sign of each coefficient indicates the direction of association with the prediction outcome, while the absolute magnitude reflects the relative contribution strength after regularization. These selected deep features were then used for subsequent RP prediction modeling.

### 3.2. Five-Fold Cross-Validation

This study employs five-fold cross-validation to evaluate the small-sample dataset. By improving data utilization, it facilitates model selection and hyperparameter tuning, thereby mitigating overfitting and reducing evaluation variance. In addition, averaging performance metrics across folds provides a more stable and reliable estimate of model performance.

**Figure 7:**
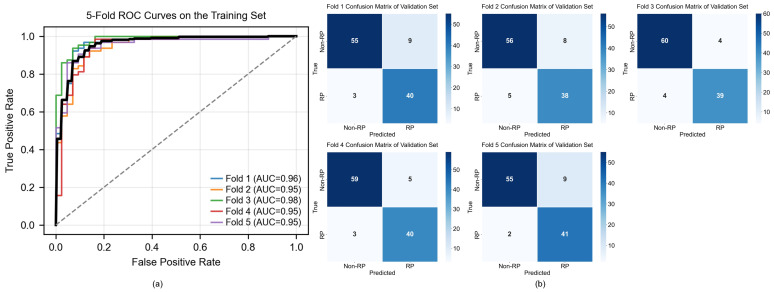
Performance of the DAAE-MIL multimodal fusion model on the training dataset using five-fold cross-validation. (**a**) ROC curves of the validation folds derived from five-fold cross-validation, indicating stable and robust performance across folds (AUC range: 0.95–0.98). The dashed line represents the performance of a random classifier (AUC = 0.5). (**b**) Confusion matrices of the five validation folds. Darker blue indicates a higher proportion of correct classifications.

Table 2 presents the 5-Fold CV performance of the training dataset and the independent test performance of the DAAE-MIL multimodal fusion model for RP prediction. The dataset was split into training and testing sets (independent test) at an 8:2 ratio, ensuring complete independence between the two. The model achieved an average AUC of 0.96 (95% CI: 0.92–0.99) on the training set and 0.97 (95% CI: 0.91–1.00) on the independent test set. Figure 7 illustrates the ROC curves and confusion matrices for each fold on the training dataset under 5-Fold CV, clearly demonstrating the predictive capability of the DAAE-MIL multimodal fusion model for identifying RP.

### 3.3. Ablation Experiment

**Figure 8:**
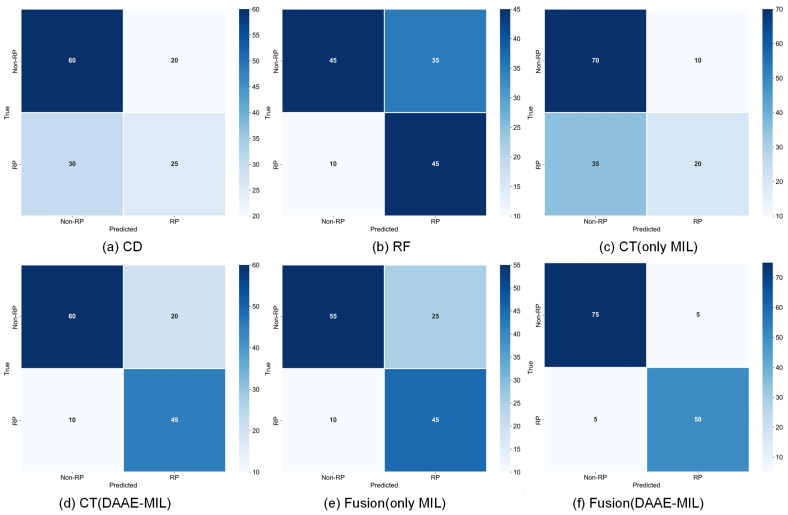
Comparison of confusion matrices in ablation experiments on the test dataset. (**a**) CD: clinical data, (**b**) RF: radiomics features, (**c**) CT (only MIL): conventional MIL feature extraction, (**d**) CT (DAAE-MIL): DAAE-MIL feature extraction, (**e**) CD + RF + CT (only MIL): fusion prediction with conventional MIL, (**f**) CD + RF + CT (DAAE-MIL): fusion prediction with DAAE-MIL.

To comprehensively evaluate the effectiveness of the proposed method, a series of ablation experiments was designed to quantify the contribution of each module to the performance improvement. As illustrated in Figure 8 and Table 3, the experimental results compare the performance of unimodal and multimodal prediction models. For the unimodal models, DAAE-MIL CT significantly outperformed MIL-only CT across all evaluation metrics, with notable improvements in accuracy (+0.11), F1-score (+0.14), and AUC (+0.10). These results confirm that the DAAE-MIL framework effectively captures critical instance-level features from CT scans, thereby enhancing the prediction of radiation pneumonitis (RP). In the multimodal setting, the DAAE-MIL fusion model achieved state-of-the-art performance, obtaining an AUC of 0.97—an improvement of 0.17 over MIL-only fusion (AUC = 0.80)—along with balanced precision (0.91), recall (0.91), and an F1-score of 0.91.Notably, it outperformed the best unimodal model (CT (DAAE-MIL)) by 13–20% across all metrics. Meanwhile, the ablation study results of the only MIL and DAAE-MIL methods demonstrate that the dual-layer attention mechanism effectively filters irrelevant noise from negative instances through key instance selection, thereby enhancing the diagnostic value of bag-level features.

Furthermore, confusion matrix analysis (Figure 8) is fully consistent with the quantitative results in Table 3. In particular, Figure 8f (fusion (DAAE-MIL)) shows the lowest total error count, with a misclassification rate of 10/135=0.074. This reduction in classification errors is simultaneously reflected by the best overall metric profile in Table 3, including accuracy (0.93), precision (0.91), recall (0.91), the F1-score (0.91), and AUC (0.97). By comparison, the MIL-only fusion model shows a substantially weaker performance (accuracy = 0.74, precision = 0.75, recall = 0.74, F1-score = 0.74, and AUC = 0.80). Collectively, these results demonstrate the proposed method’s effectiveness in distinguishing RP from non-RP cases with high predictive accuracy and robustness.

### 3.4. Visualization Analysis of Critical Instance Selection in DAAE

**Figure 9:**
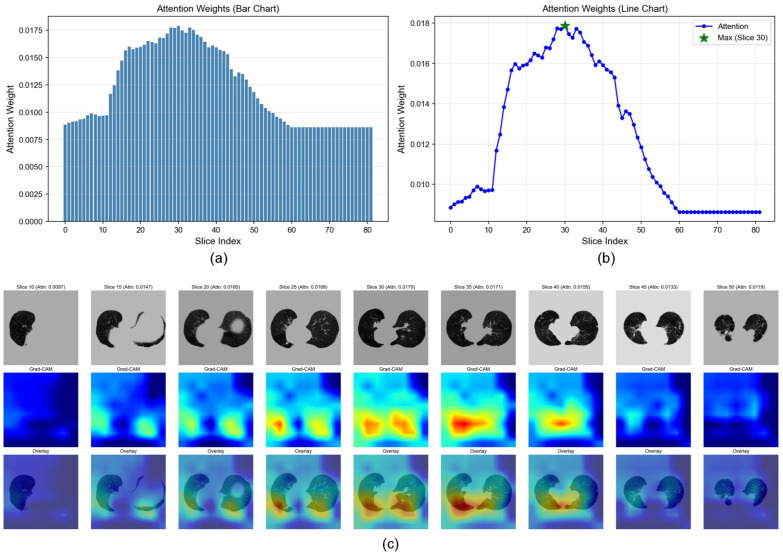
Visualization of attention-based critical slice selection and interpretability analysis in the DAAE framework. (**a**) Attention weight distribution across all CT slices illustrated by a bar chart. (**b**) Line plot of attention weights, where the slice with the maximum attention score is highlighted. (**c**) Representative CT slices with different attention scores and their corresponding Grad-CAM class activation maps and overlay visualizations, demonstrating that slices with higher attention scores focus on more discriminative lung regions. In the heatmaps, red indicates high-attention regions, and blue indicates low-attention regions.

To evaluate the performance of the DAAE framework in selecting critical slices from pre-radiotherapy lung CT images (Lung-PTV), Grad-CAM was employed to provide an interpretability analysis of the instance selection process. Specifically, DAAE selects slices with higher attention scores and embeds them into instance bags, thereby improving the accuracy of deep feature extraction from CT images. As shown in Figure 9, the attention scores of all CT slices and the corresponding class activation maps over the selected slice ranges are presented, which partially alleviates the interpretability limitations of deep feature extraction.

## 4. Discussion

RP is among the most serious complications experienced by lung cancer patients undergoing radiotherapy. Its pathogenesis is complex and influenced by multiple factors—including the radiation dose, baseline pulmonary condition, and prior medical history—which collectively complicate clinical prediction and prevention [23]. To address this challenge, we propose a multimodal fusion prediction model based on DAAE-MIL, which integrates clinical variables (e.g., dosimetric parameters), radiomics features, and deep representations extracted from CT scans. The experimental results show that the multimodal fusion methods achieve AUCs of 0.80 and 0.97, substantially outperforming single-modal models (the best of which, DAAE-MIL CT, achieved an AUC of 0.77) and highlighting the value of data fusion in RP prediction. Using MIL-only fusion as the baseline, DAAE-MIL fusion achieves significantly better performance across all metrics (*p* < 0.001), demonstrating the effectiveness of the DAAE module in enhancing RP prediction.

Table 4 summarizes and compares existing methods for RP prediction. Due to the heterogeneity in data modalities and implementation details across different RP prediction methods, we only compare our results with the experimental results reported in the cited studies. The baseline models involved have been presented in Table 3 and Table 5. Hirose et al. [24] employed unimodal radiomics features without incorporating clinical variables, which may limit the model’s generalizability. In contrast, multimodal learning enables the integration of heterogeneous patient data, thereby overcoming the inherent limitations of unimodal strategies. Although Zhang et al. [8] and Luna et al. [25] also utilized multimodal fusion, their predictive performance suffered due to the vulnerability of their models to irrelevant instances caused by suboptimal feature extraction. Nie et al. [26] combined dosimetric data and clinical and radiomics features to predict symptomatic RP in patients undergoing combined immunotherapy and radiotherapy, using a ResNet-based approach to extract features from CT ROIs, including the gross tumor volume (GTV), PTV, and the surrounding area between PTV and GTV. Although CT scans encompass multiple ROIs and exhibit improved predictive performance, the features extracted by ResNet are susceptible to contamination from non-critical information, thereby compromising the stability of the predictions. The method proposed by Nie et al. [26] achieves slightly lower performance compared to the results reported in their original paper, which is attributed to differences in datasets. Specifically, this study only uses the Lung-PTV region as the ROI in CT, whereas the original study employed multiple ROI regions. In this study, we introduce a DAAE-MIL-based model that employs a fine-tuned C3D network to extract deep features from CT images. These features are subsequently processed through the DAAE-MIL framework, which identifies highly relevant instances and embeds them into virtual bags to capture discriminative instance-level patterns. By integrating clinical data, radiomics features, and deep CT representations, our model achieves an AUC of 0.97—surpassing existing approaches—and maintains robust performance even when the region of interest is limited to Lung-PTV, and this reduction in ROI selection lowers research costs while maintaining comparable predictive performance.

**Figure 10:**
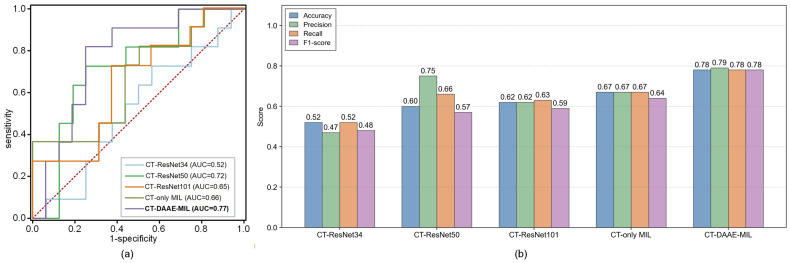
Performance comparison of CT deep feature extraction models. (**a**) ROC curves demonstrate the diagnostic efficacy of five methods: CT-ResNet34 (AUC = 0.52), CT-ResNet50 (AUC = 0.72), CT-ResNet101 (AUC = 0.65), CT-only MIL (AUC = 0.66), and CT-based DAAE-MIL (AUC = 0.77). The dashed line represents the performance of a random classifier (AUC = 0.5). (**b**) Comparative metrics of five methods.

Notably, the CT-derived deep features capture latent pathological factors associated with RP onset. Compared to the ResNet-based feature extractor used by Nie et al. [26], our model exhibits superior performance, as detailed in Table 5. As shown in Figure 10, the ROC curves for different feature extractors reveal that among ResNet variants, ResNet50 achieves the best performance (AUC = 0.72), though it is still approximately 5% lower than that of the proposed DAAE-MIL model. Moreover, the DAAE-MIL model consistently outperforms ResNet50 across all classification metrics, confirming its advantage in RP prediction. However, due to the black-box nature of deep learning, the extracted features cannot be directly interpreted. Nevertheless, as shown in Figure 6, the 21 deep features outperform the 15 handcrafted radiomics features presented in Figure 5 in terms of predictive performance (see Table 3). Specifically, the CT (DAAE-MIL) model achieves an AUC of 0.77 (+0.08) and an accuracy of 0.78 (+0.11) over the handcrafted features. This also validates that the deep features extracted by DAAE-MIL improve prediction accuracy.

In summary, our proposed model demonstrates high predictive accuracy and holds substantial clinical relevance in RP risk assessment. It can assist clinicians in identifying high-risk patients before radiotherapy, facilitating personalized treatment adjustments such as radiation dose reduction. Furthermore, the model enables early intervention for patients likely to develop RP, potentially reducing its incidence and improving overall patient outcomes. Despite the achievements of this study, there are still certain limitations in RP prediction that require further improvements in future research. First, the retrospective nature of the dataset and the fact that data were sourced from a single center may introduce potential cohort biases and limit the generalizability of the prediction model. Future work will collect multicenter data to enhance the model’s generalizability and stability. Second, the clinical data used in this study include only basic clinical parameters and radiation dose data, without considering additional clinical information (such as lung function indicators and biological factors) and dose distribution maps. Future research will expand the feature set and increase the sample size to improve the prediction accuracy and robustness of the model.

## 5. Conclusions

This paper proposes a multimodal fusion-based DAAE-MIL prediction model for RP. The model first extracts deep features from CT images using a fine-tuned C3D model and employs the DAAE-MIL method to filter high-relevance instances, embedding them into instance bags to extract key instance deep features. Subsequently, clinical data, radiomics features, and CT deep features selected by LASSO regression are integrated to achieve accurate RP prediction. Experimental results demonstrate that the model exhibits significant predictive performance across multiple validation tests, with superior results in various metrics, including an ACC of 0.93 and an AUC of 0.97, both of which are markedly better than those of unimodal methods. Furthermore, this study suggests that the DAAE-MIL model demonstrates potential clinical value in RP prediction, providing a promising risk assessment tool for lung cancer patients undergoing radiotherapy and assisting clinicians in optimizing treatment plans.

## Figures and Tables

**Table 1:** Correlation analysis of clinical data.

Characteristics	Non-RP	RP	*p*-Value
Age			0.48
≥65	185	110	
<65	215	160	
Smoking			0.93
Yes	155	125	
No	245	145	
Chemotherapy			0.82
Yes	285	160	
No	115	110	
Histology			0.68
SCC ^1^	180	80	
Non-SCC	220	190	
T stage			0.85
1–2	255	180	
3–4	145	90	
N stage			0.28
0–1	140	70	
2–3	260	200	
Lung dose–volume metrics (mean ± SD)			
Lung–V5	43.75 ± 13.65	46.43 ± 14.88	0.005
Lung–V10	31.15 ± 10.25	33.34 ± 11.61	0.006
Lung–V20	20.09 ± 7.38	21.86 ± 8.67	0.002
Lung–V30	13.17 ± 5.22	14.51 ± 6.53	<0.001
Lung–Dmean	10.82 ± 3.40	11.63 ± 4.26	<0.001

^1^ SCC: squamous cell carcinoma, SD: standard deviation.

**Table 2:** Performance of the DAAE-MIL multimodal fusion model evaluated by five-fold cross-validation on the training dataset and independent testing on the test dataset.

Dataset	Fold	Accuracy	Precision	Recall	F1-Score	AUC (CI: 95%)
Train (5-Fold Validation)	Fold 1	0.89	0.95	0.86	0.90	0.96 (0.92, 0.99)
	Fold 2	0.88	0.92	0.88	0.90	0.95 (0.90, 0.98)
	Fold 3	0.93	0.94	0.94	0.94	0.98 (0.96, 1.00)
	Fold 4	0.93	0.95	0.92	0.94	0.96 (0.90, 0.99)
	Fold 5	0.90	0.96	0.86	0.91	0.95 (0.91, 0.99)
	Mean	0.91	0.94	0.89	0.92	0.96 (0.92, 0.99)
Test (Independent Testing)	/	0.93	0.91	0.91	0.91	0.97 (0.91, 1.00)

**Table 3:** Comparative results of ablation experiments on the test dataset.

Experiments	Accuracy	Precision	Recall	F1-Score	AUC	*p*-Value
CD ^1^	0.63	0.61	0.60	0.60	0.73	<0.001
RF ^2^	0.67	0.71	0.67	0.67	0.69	0.002
CT (only MIL) ^3^	0.67	0.67	0.67	0.64	0.66	<0.001
CT (DAAE-MIL) ^4^	0.78	0.79	0.78	0.78	0.77	0.021
Fusion (only MIL) ^5^	0.74	0.75	0.74	0.74	0.80	–
Fusion (DAAE-MIL) ^6^	**0.93**	**0.91**	**0.91**	**0.91**	**0.97**	**<0.001**

^1^ CD: clinical data, ^2^ RF: radiomics features, ^3^ CT: CT deep features, ^4^ only MIL: multiple-instance learning, ^5^ DAAE-MIL: dual-layer attention-based adaptive bag embedding MIL, ^6^ Fusion: CD + RF + CT. – indicates the reference (baseline) model row for *p*-value comparison. The best values are shown in bold.

**Table 4:** Comparison of RP prediction research results.

Reference	Features	Methods	AUC
Hirose et al. [24]	Radiomics features	Logistic Regression	0.76
Zhang et al. [8]	CT + Radiomics features	CNN	0.83
Luna et al. [25]	Dosimetric data + Clinical	Random Forest	0.66
Nie et al. [26]	Dosimetric + Clinical + Radiomics	MLP (ResNet50) ^1^	0.95
This paper	CT + Radiomics + Clinical (Dosimetric)	MLP (DAAE-MIL) ^2^	**0.97**

^1^ MLP(ResNet50): deep feature extraction using the ResNet50 architecture, with subsequent prediction model construction via MLP, ^2^ MLP(DAAE-MIL): deep feature extraction using the DAAE-MIL architecture, with subsequent prediction model construction via MLP. The best values are shown in bold.

**Table 5:** The DAAE-MIL extracts the CT deep feature performance.

Method	Accuracy	Precision	Recall	F1-Score	AUC	*p*-Value
CT-ResNet34	0.52	0.47	0.52	0.48	0.52	–
CT-ResNet50	0.60	0.75	0.66	0.57	0.72	0.002
CT-ResNet101	0.62	0.62	0.63	0.59	0.65	0.008
CT-only MIL	0.67	0.67	0.67	0.64	0.66	<0.001
CT-DAAE-MIL	**0.78**	**0.79**	**0.78**	**0.78**	**0.77**	**<0.001**

– indicates the reference (baseline) model row for *p*-value comparison. The best values are shown in bold.

## Data Availability

The data presented in this study are available on request from the corresponding author due to privacy and ethical restrictions.

## References

[1] Bray F., Laversanne M., Sung H., Ferlay J., Siegel R., Soerjomataram I., Jemal A. (2024). Global cancer statistics 2022: GLOBOCAN estimates of incidence and mortality worldwide for 36 cancers in 185 countries. CA Cancer J. Clin..

[2] Simone C. (2017). Thoracic radiation normal tissue injury. Semin. Radiat. Oncol..

[3] Ullah T., Patel H., Pena G., Shah R., Fein A. (2020). A contemporary review of radiation pneumonitis. Curr. Opin. Pulm. Med..

[4] Li P., Zhang J., Tian X. (2023). Radiation Pneumonia. Radiology of Infectious and Inflammatory Diseases-Volume 3: Heart and Chest.

[5] Zhang H., Tan S., Chen W., Kligerman S., Kim G., D’Souza W., Suntharalingam M., Lu W. (2014). Modeling pathologic response of esophageal cancer to chemoradiation therapy using spatial-temporal 18F-FDG PET features, clinical parameters, and demographics. Int. J. Radiat. Oncol. Biol. Phys..

[6] Raptis S., Softa V., Angelidis G., Ilioudis C., Theodorou K. (2023). Automation Radiomics in Predicting Radiation Pneumonitis (RP). Automation.

[7] Lee J., Kang M., Park J., Lee S.J., Kim J.C., Park S.H. (2024). Deep-Learning Model Prediction of Radiation Pneumonitis Using Pretreatment Chest Computed Tomography and Clinical Factors. Technol. Cancer Res. Treat..

[8] Zhang Z., Wang Z., Luo T., Yan M., Dekker A., De Ruysscher D., Traverso A., Wee L., Zhao L. (2023). Computed tomography and radiation dose images-based deep-learning model for predicting radiation pneumonitis in lung cancer patients after radiation therapy. Radiother. Oncol..

[9] Kawahara D., Imano N., Nishioka R., Nagata Y. (2023). Image masking using convolutional networks improves performance classification of radiation pneumonitis for non-small cell lung cancer. Phys. Eng. Sci. Med..

[10] Ai Y., Ni W., Su W., Jin X., Shen Y., Huang W., Xiang Z., Yu X., Xie C., Jin X. (2025). Integrating deep learning and multi-omics features in radiation pneumonitis prediction for lung cancer patients using PET/CT. BMC Med. Imaging.

[11] Kong Y., Su M., Zhu Y., Li X., Zhang J., Gu W., Yang F., Zhou J., Ni J., Yang X. (2025). Enhancing the prediction of symptomatic radiation pneumonitis for locally advanced non-small-cell lung cancer by combining 3D deep learning-derived imaging features with dose–volume metrics: A two-center study. Strahlenther. Onkol..

[12] Chen Y., Bi J., Wang J. (2006). MILES: Multiple-instance learning via embedded instance selection. IEEE Trans. Pattern Anal. Mach. Intell..

[13] Raykar V., Krishnapuram B., Bi J., Dundar M., Rao R. (2008). Bayesian multiple instance learning: Automatic feature selection and inductive transfer. Proceedings of the 25th International Conference on Machine Learning.

[14] Kandemir M., Hamprecht F. (2015). Computer-aided diagnosis from weak supervision: A benchmarking study. Comput. Med. Imaging Graph..

[15] Dietterich T., Lathrop R., Lozano-Pérez T. (1997). Solving the multiple instance problem with axis-parallel rectangles. Artif. Intell..

[16] Zhang Q., Goldman S. (2001). EM-DD: An improved multiple-instance learning technique. Adv. Neural Inf. Process. Syst..

[17] Andrews S., Tsochantaridis I., Hofmann T. (2002). Support vector machines for multiple-instance learning. Adv. Neural Inf. Process. Syst..

[18] Zhu L., Zhao B., Gao Y. (2008). Multi-class multi-instance learning for lung cancer image classification based on bag feature selection. Proceedings of the 2008 Fifth International Conference on Fuzzy Systems and Knowledge Discovery, Jinan, China.

[19] Xu Y., Zhu J., Eric I., Lai M., Tu Z. (2014). Weakly supervised histopathology cancer image segmentation and classification. Med. Image Anal..

[20] Li C., Shi C., Zhang H., Chen Y., Zhang S. (2015). Multiple instance learning for computer aided detection and diagnosis of gastric cancer with dual-energy CT imaging. J. Biomed. Inform..

[21] Li Z., Yuan L., Xu H., Cheng R., Wen X. (2020). Deep multi-instance learning with induced self-attention for medical image classification. Proceedings of the 2020 IEEE International Conference on Bioinformatics and Biomedicine (BIBM), Seoul, Republic of Korea.

[22] Cheplygina V., Tax D., Loog M. (2015). Multiple instance learning with bag dissimilarities. Pattern Recognit..

[23] McDonald K.E., Morrow C.J., DiNardo L.B., Chen Y.C., Chen L., Yu J.B., Shen R., Bass A.R., Chen M., Zhang Q. (2023). Predictors of pneumonitis in patients with locally advanced non-small cell lung cancer treated with definitive chemoradiation followed by consolidative durvalumab. Adv. Radiat. Oncol..

[24] Hirose T., Arimura H., Ninomiya K., Yoshitake T., Fukunaga J., Shioyama Y. (2020). Radiomic prediction of radiation pneumonitis on pretreatment planning computed tomography images prior to lung cancer. Sci. Rep..

[25] Luna J., Chao H., Diffenderfer E., Valdes G., Chinniah C., Ma G., Cengel K., Solberg T., Berman A., Simone C. (2019). Predicting radiation pneumonitis in locally advanced stage II–III non-small cell lung cancer using machine learning. Radiother. Oncol..

[26] Nie T., Chen Z., Cai J., Ai S., Xue X., Yuan M., Li C., Shi L., Liu Y., Verma V. (2024). Integration of dosimetric parameters, clinical factors, and radiomics to predict symptomatic radiation pneumonitis in lung cancer patients undergoing combined immunotherapy and radiotherapy. Radiother. Oncol..

